# Estimation of genetic parameters and detection of quantitative trait loci for minerals in Danish Holstein and Danish Jersey milk

**DOI:** 10.1186/s12863-015-0209-9

**Published:** 2015-05-21

**Authors:** Bart Buitenhuis, Nina A Poulsen, Lotte B Larsen, Jakob Sehested

**Affiliations:** Aarhus University, Center for Quantitative Genetics and Genomics, Department of Molecular Biology and Genetics, Blichers Allé 20, P.O. Box 50, DK-8830 Tjele, Denmark; Aarhus University, Department of Food Science, Blichers Allé 20, P.O. Box 50, DK-8830 Tjele, Denmark; Aarhus University, Department of Animal Science, Blichers Allé 20, P.O. Box 50, DK-8830 Tjele, Denmark

**Keywords:** Bovine milk, Minerals, Element, Genetic parameters, Association study

## Abstract

**Background:**

Bovine milk provides important minerals, essential for human nutrition and dairy product quality. For changing the mineral composition of the milk to improve dietary needs in human nutrition and technological properties of milk, a thorough understanding of the genetics underlying milk mineral contents is important. Therefore the aim of this study was to 1) estimate the genetic parameters for individual minerals in Danish Holstein (DH) (n = 371) and Danish Jersey (DJ) (n = 321) milk, and 2) detect genomic regions associated with mineral content in the milk using a genome-wide association study (GWAS) approach.

**Results:**

For DH, high heritabilities were found for Ca (0.72), Zn (0.49), and P (0.46), while for DJ, high heritabilities were found for Ca (0.63), Zn (0.57), and Mg (0.57). Furthermore, intermediate heritabilities were found for Cu in DH, and for K, Na, P and Se in the DJ. The GWAS revealed a total of 649 significant SNP markers detected for Ca (24), Cu (90), Fe (111), Mn (3), Na (1), P (4), Se (12) and Zn (404) in DH, while for DJ, a total of 787 significant SNP markers were detected for Ca (44), Fe (43), K (498), Na (4), Mg (1), P (94) and Zn (3). Comparing the list of significant markers between DH and DJ revealed that the SNP ARS-BFGL-NGS-4939 was common in both breeds for Zn. This SNP marker is closely linked to the DGAT1 gene. Even though we found significant SNP markers on BTA14 in both DH and DJ for Ca, and Fe these significant SNPs did not overlap.

**Conclusion:**

The results show that Ca, Zn, P and Mg show high heritabilities. In combination with the GWAS results this opens up possibilities to select for specific minerals in bovine milk.

**Electronic supplementary material:**

The online version of this article (doi:10.1186/s12863-015-0209-9) contains supplementary material, which is available to authorized users.

## Background

Bovine milk provides essential substances for growth and development of the newborn. Besides water, milk consists of proteins, fat, lactose, vitamins and minerals, in addition to other components like metabolites and free oligosaccharides. The mineral fraction constitutes a minor fraction of the milk solids (approximately 7.1 to 7.4 g/L), and comprises cations (among others calcium (Ca), magnesium (Mg), sodium (Na) and potassium (K)), and anions (among others phosphorus, and chloride) [1,2]. Minerals contribute to important physiological processes and in human nutrition it has been shown that e.g. Ca and P play a role in bone metabolism, Se and Zn play a role in the immune system while Ca, K and Mg are involved in maintaining blood pressure [3-5]. High mineral content and availability of milk makes dairy products important sources of minerals to the human diet [6,7].

Furthermore, the mineral composition of milk is important for the technological properties of milk, because minerals are involved in the structure and stability of casein micelles (micellar bound) and thereby e.g. the coagulation properties of the milk. Free divalent cations, especially Ca ions in milk serum, significantly influence the surrounding environment of the negatively charged casein micelles [8] and thereby the coagulation properties of the milk.

For changing the mineral composition of milk to improve dietary needs in human nutrition or to improve technological properties of milk, two different strategies can be employed: Fortification or increasing the natural content. As fortification by e.g. Ca is problematic for different reasons, including changing the milk pH and stability [9] a good understanding of the genetics underlying milk mineral content is needed in relation to explore the possibilities for increasing mineral content through feeding or breeding. It has been shown that the mineral content in bovine milk is influenced by non-genetic factors like lactation stage and nutrition [3,10]. However, substantial genetic variation for a number of minerals in bovine milk has been reported [11]. In the current study we have collected tissue and milk samples from Danish Holstein and Danish Jersey dairy cows. The tissue samples were examined using the bovine 777 k single nucleotide polymorphism (SNP) chip combined with detailed milk mineral profiling by inductively coupled plasma mass spectrometry (ICP-MS). The aim of this study was to 1) estimate the genetic parameters for individual minerals in Danish Holstein (DH) and Danish Jersey (DJ) dairy milk, and 2) detect genomic regions associated with mineral content in the milk using a genome-wide association study (GWAS) approach.

## Methods

### Animals

All procedures were performed in accordance with the National Guidelines for Animal Experimentation and the guidelines of the Danish Animal Experimental Ethics Committee. Within the Danish-Swedish Milk Genomics Initiative, morning milk samples were collected from 456 DH cows (20 dairy herds, October - December 2009) and 436 DJ (22 dairy herds, February – April 2010). From each herd, between 19 and 24 cows were sampled. All cows were housed in loose housing systems, fed according to standard practice, and milked twice a day. The cows sampled were all in mid-lactation (d129 to d229 in DH and d130 to d252 in DJ) and within parity 1 to 3. Immediately after milking, milk samples were placed on ice for transport to the laboratory. Once at the laboratory, the milk samples were treated as described by Poulsen et al. [12].

### Micro and macro elements

Ten different elements (Ca, Cu, Fe, K, Mg, Mn, Na, P, Se, and Zn) were extracted from skimmed milk by acid sonication and identified using inductively coupled plasma mass spectrometry (ICP-MS) as described by Cava-Montesinos et al. [13].

References and standards were analyzed in parallel with the skimmed milk samples. The reference was whole milk powder 1549a from The National Institute of Standards and Technology (NIST, U.S. Department of Commerce). For Ca, Mg, P, S, Na and K the standard CZ9097 Mix 017 from the Czeck Metrology Institute (Analytika Ltd., Praque, Czeck Republic) was used. For Cu, Fe, Mn, Se, and Zn, the standard PlasmaCAL custom standard from SCP Science, Canada, was used.

Samples were heated to 40°C in a water bath. Two g of each milk sample was diluted with 2 mL aqua regia (65% super pure nitric acid and 30% super pure hydrochloric acid) in 13 mL polystyrene tubes (Deltalab, Spain), shaken for a few seconds on Whirl-mixer and left until next day. Samples were then ultrasonicated (Branson 3510, Danbury, USA) for 10 minutes and diluted to 10 mL by 2% super-pure nitric acid, and ultrasonicated for another 10 minutes before centrifugation at 3,578 *g* for 2 minutes. The supernatant (5 mL) was transferred to new vials 13 mL polystyrene tubes (Deltalab, Spain) and analyzed by ICP-MS (X-series II) from Thermo Fisher Scientific Inc., U.S.

### SNP chip and genotyping

The genotyping procedure was carried out as described earlier [14]. In short, 371 DH and 321 DJ cows were genotyped with the bovineHD beadchip [15]. The bovineHD chip contains 777,962 markers with a median interval of 2.68 kb between SNPs (www.illumina.com/documents/products/datasheets/datasheet_bovineHD.pdf). Genomic DNA was extracted from ear tissue. The platform used was an Illumina® Infinium II Multisample assay device. SNP chips were scanned using iScan and analyzed using Beadstudio version 3.1 software. The quality parameters used for the selection of SNPs in the GWAS were minimum call rates of 80% for individuals and 95% for loci. Marker loci with minor allele frequencies (MAFs) below 1% were excluded. The quality of the markers was assessed using the GenCall data analysis software of Illumina. Individuals with average GenCall scores below 0.65 were excluded following Teo et al. [16]. The SNP positions were based on the *Bos taurus* genome assembly (*Btau_4.0*) [17]. In total 494,984 SNP markers were used in both DH and DJ.

### SNPs assigned to genes

For each gene known in the cattle database, location in the bovine genome was determined as 5Kb before the start position of the first exon to 5Kb after the end position of the last exon. Hence, the defined gene region includes all introns and parts of the upstream and downstream regions of the gene. When a SNP was located in this region it was assigned to the corresponding gene.

### Statistical analysis

The statistical analysis was based on the 315 DH and 316 DJ animals having both genotypic and phenotypic records.

### Calculation of the G-matrix

The calculation of the genomic relationship matrix has been described in detail by Buitenhuis et al. [14]. In short, the genomic relationship matrix was calculated for each breed separately. For each chromosome, a genomic relationship matrix as described by the first method presented in VanRaden [18] was calculated as follows: Let **M** be a matrix with dimensions of the number of individuals (*n*) by the number of loci (*m*) that specifies which marker alleles each individual inherited. The elements of **M** were set to −1, 0, 1 for the homozygote, heterozygote and the other homozygote, respectively. The diagonals of **M’M** counts the number of homozygous loci for each individual and off diagonals measure the number of alleles shared by relatives. Let the frequency of the second allele at locus *i* be *p*_*i*_, and let **P** contain the allele frequencies, such that column *i* of **P** equals 2(*p*_*i*_-0.5). Subtraction of **P** from **M** gives **Z**, which is needed to set the expected mean value to 0. The genomic relationship matrix **G** was then calculated as **ZZ´**/[2∑p_i_(1-p_i_)] [18].

### Estimation of heritability

To estimate the genetic parameters and variance components the REML approach in DMU was used [19]. The following model was used in the analysis:

1 $$ {\mathrm{Y}}_{\mathrm{i}\mathrm{jkl}}=\upmu +{\mathrm{herd}}_{\mathrm{i}}+{\mathrm{parity}}_{\mathrm{j}}+{\mathrm{b}}_1\times {\mathrm{DIM}}_{\mathrm{k}}+{\mathrm{animal}}_{\mathrm{l}}+{\mathrm{e}}_{\mathrm{i}\mathrm{jkl}} $$

Where Y_ijkl_ is the phenotype of individual l in herd i and lactation j, μ is the fixed mean effect, herd_i_ is a fixed effect (i = 1, 2, … , 19 DH; i = 1, 2, … , 22 DJ), parity_j_ is a fixed effect (j = 1, 2, 3 DH, j = 1, 2, 3 DJ), b_1_ is the regression coefficient for DIM_k_, DIM_k_ is a covariate of days in milk (d129 to d229 in DH, d130 to d252 in DJ), and animal is the random additive genetic effect based on **G** of animal l [20].

The milk samples were taken once on each farm in a short period while the cows were housed inside, therefore we did not fit a season effect in the model.

Univariate analyses were performed to estimate the heritability, which was defined as:

2 $$ {\mathrm{Y}}_{\mathrm{i}\mathrm{jkl}}=\upmu +{\mathrm{herd}}_{\mathrm{i}}+{\mathrm{parity}}_{\mathrm{j}}+{\mathrm{b}}_1\times {\mathrm{DIM}}_{\mathrm{k}}+{\mathrm{animal}}_{\mathrm{l}}+{\mathrm{e}}_{\mathrm{i}\mathrm{jkl}} $$

where σ^2^_a_ was the genetic variation, and σ^2^_e_ was the residual variation.

### Association mapping

The association analysis was performed using the following linear model for each breed separately:

3 $$ {\mathrm{h}}^2={\upsigma^2}_{\mathrm{a}}/\left({\sigma^2}_{\mathrm{a}}+{\sigma^2}_{\mathrm{e}}\right) $$

Where Y_ijklm_ is the phenotype of individual l in herd i and lactation j, μ is the fixed mean effect, herd_i_ is a fixed effect (i = 1, 2, … , 19 DH; i =1, 2, … , 22 DJ), parity_j_ is a fixed effect (j = 1, 2, 3 DH, j = 1, 2, 3 DJ), b_1_ is the regression coefficient for DIM_k_, DIM_k_ is a covariate of days in milk (d129 to d229 in DH, d130 to d252 in DJ), b_2_ is the allele substitution effect, SNP_m_ is a covariate indicating if a SNP is homozygote (0,2) or heterozygote (1), and animal is the random additive genetic effect based on **G** of animal l [20]. The effect of the SNP was tested by a Wald test with a null hypothesis of H_0_: b = 0. The analyses were performed using REML in the R interface of DMU [19] (available at *http://dmu.agrsci.dk*). Significance thresholds were determined using a false discovery rate (FDR) correction using the R package “qvalue” version 1.34.0. A FDR of P < 0.10 was considered significant.

### Linkage disequilibrium

The LD block around the most significant marker in a QTL was determined using HAPLOVIEW [21].

## Results

In Table 1, the phenotypic mean, standard deviations and CVs for the minerals in the milk of DH and DJ are given. In general, DH has a lower mineral content (Ca, Cu, Fe, Mg, Mn, Na, P, Se, and Zn) in the milk compared to DJ, except for K which is higher in the DH (1469.8 ppm ± 115.0 DH vs. 1319.0 ppm ±104.9 DJ). The mean levels of minerals in DH milk were in the range of 0.007 ppm for Se to 1469.8 ppm for K, whereas the range for minerals in DJ milk was from 1468.8 ppm for Ca to 0.01 ppm for Se. The CVs were in the range of 7.8% for K to 33.3% for Cu in DH, and 7.9% for K to 40.0% for Cu in DJ.

**Table 1:** **Mean (ppm) and standard deviation of micro and macro elements in Danish Holstein and Danish Jersey milk**

**Trait**	**Danish Holstein**			**Danish Jersey**		
	**Mean** ^**1**^	**SD**	**CV (%)**	**Mean** ^**1**^	**SD**	**CV (%)**
Ca	1214.2	123	10.1	1465	148	10.1
K	1470	115	7.8	1319	105	7.9
Na	349	74	21.1	389	101	25.9
P	725	78	10.8	880	93	10.6
Mg	108	11	9.9	124	13	10.3
Cu	0.03	0.01	33.3	0.05	0.02	40.0
Fe	0.17	0.04	23.5	0.19	0.05	26.3
Mn	0.02	0.005	25.0	0.03	0.009	30.0
Se	0.007	0.002	28.6	0.01	0.002	20.0
Zn	3.39	0.63	11.5	4.73	0.79	16.7

^1^the mean of all micro and macro elements for the Danish Holstein differ significantly (P < 0.001) from the Danish Jersey.

Phenotypic correlations between the mineral and overall milk composition show similarity in both the DH as well as the DJ (Additional file 1: Table S1). Especially P, Ca and Mg were positively correlated and further showed a strong correlation to protein content. In cases there are differences in the sign of the correlation, these correlations are not significant, i.e. the standard error is much larger compared to the estimate. However there is one exception: Lactose and K has a negative correlation (−0.22 ± 0.06) in the DH, while these components have a positive correlation (0.17 ± 0.06) in DJ (Additional file 1: Table S1).

The heritability estimates and the genetic variance for the minerals for both DH and DJ are presented in Table 2. For DH, high heritabilities were found for Ca (0.72), Zn (0.49), and P (0.46). For DJ, high heritabilities were found for Ca (0.63), Zn (0.57), and Mg (0.57). Intermediate heritabilities were found for Cu in DH, and for K, Na, P and Se in the DJ (Table 2).

**Table 2:** **Additive genetic variance (σ** ^**2**^_**A**_ **), heritability (h** ^**2**^ **) for Ca, Cu, Fe, K, Mg, Mn, Na, P, Se, and Zn in both the Danish Holstein and Danish Jersey**

**Trait**	**DH σ** ^**2**^_**A**_	**DH h** ^**2**^	**DJ σ** ^**2**^_**A**_	**DJ h** ^**2**^
Ca	10278.18	0.72 (0.36)	13017.58	0.63 (0.31)
K	NC^1^	NC^1^	3125.03	0.32 (0.21)
Na	NC^1^	NC^1^	1406.43	0.20 (0.19)
P	2547.80	0.46 (0.25)	2260.37	0.29 (0.21)
Mg	7.13	0.08 (0.21)	94.91	0.57 (0.25)
Cu	5.31E-05	0.28 (0.25)	NC^1^	NC^1^
Fe	2.61E-07	1.91E-4 (0.22)	4.02E-4	0.15 (0.19)
Mn	3.05E-06	0.13 (0.17)	NC^1^	NC^1^
Se	1.99E-07	0.10 (0.19)	4.73E-07	0.20 (0.14)
Zn	0.19	0.49 (0.24)	0.33	0.57 (0.22)

(SE of the estimates in parenthesis).

^1^NC: The model is not converging due to the fact that the genetic variance component goes to zero.

### GWAS results

The results of the GWAS are presented in Additional file 1: Table S1 for DH and Additional file 2: Table S2 for DJ including the allele-substitution effect, location and annotation.

### Danish Holstein

In total 649 significant SNP markers were detected for Ca (24), Cu (90), Fe (111), Mn (3), Na (1), P (4), Se (12) and Zn (404) (Additional file 2: Table S2).

**Figure 1:**
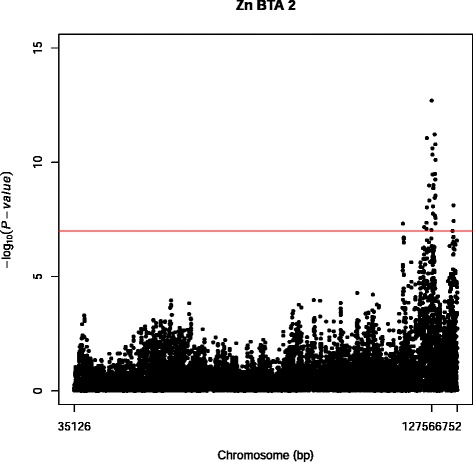
Manhattan plot of a GWAS on BTA02 for Zn for the Danish Holstein. The Y-axes show –log_10_(P-value) of single-marker associations tests. The X-axes show marker positions in base-pairs. Red horizontal line is the significance threshold at FDR < 0.10.

For Ca SNP markers were detected on BTA14. For Cu the QTL were distributed over 12 different chromosomes: BTA3 (6), BTA5 (26), BTA7 (25) and BTA14 (18) harbored most significant SNP markers. For Se 12 significant SNP markers were located on BTA8. All these 12 SNP markers were located in the same LD block (BOVINEHD0800024680-BOVINE0800024709) and assigned to chromosome 8 open reading frame, human C9orf3 *(C8H9orf3)* in the range of 82,922,191- 82,965,051 bp (BOVINEHD0800024693 - BOVINEHD0800024709). For Zn a strong QTL peak was detected on BTA2, where the most significant SNP marker (BOVINEHD0200037025) with a –log_10_(P-value) of 12.69 was assigned to PDLIM1 interacting kinase 1 like *(PDIK1L)* (Figure 1). BOVINEHD0200037025 is located in a LD block (BOVINEHD0200037022 – BOVINEHD0200037028). The gene *PDIK1L* was the only gene located in this LD block.

### Danish Jersey

In total 787 significant SNP markers were detected for Ca (44), Fe (43), K (498), Na (4), Mg (1), P (94) and Zn (3) (Additional file 3: Table S3).

**Figure 2:**
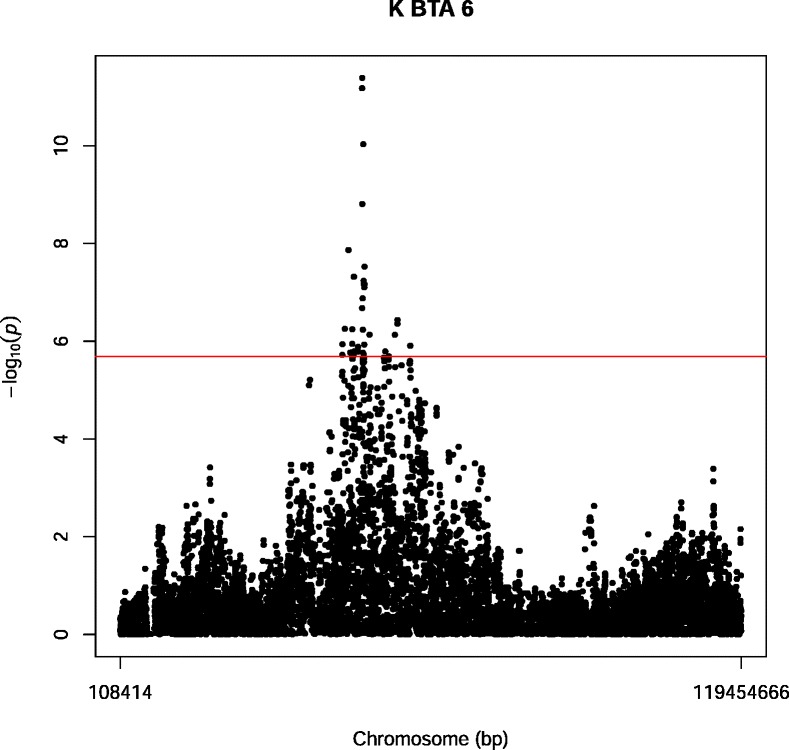
Manhattan plot of a GWAS on BTA06 for K for the Danish Jersey. The Y-axes show –log_10_(P-value) of single-marker associations tests. The X-axes show marker positions in base-pairs. Red horizontal line is the significance threshold at FDR < 0.10.

For Ca SNP markers were detected on BTA14. For Fe a QTL was detected on BTA2, and the most significant SNP marker (BOVINEHD0200029849) had a –log_10_(P-value) = 10.81 and explained 4% of the total variance. The most significant marker was located in an LD block from BOVINEHD0200029809 – BOVINEHD0200029858. For K a QTL was detected on BTA6 with the most significant SNP marker was HAPMAP23226-BTA-159656 (46,599,570 bp) which had a –log_10_(P-value) = 11.39 and explained 10.4% of the total variation (Figure 2). HAPMAP23226-BTA-159656 was located in a LD block from BOVINEHD0600012674 – BOVINEHD0600012692. There were no genes located in this LD block. For P a QTL was detected on BTA1 where the most significant SNP marker was BOVINEHD0100041584 at 144,353,573 bp with a –log_10_(P-value) = 8.79 and explained 7.8% of the total variation. The most significant marker is located in a large LD block (BOVINEHD0100041525 – BOVINEHD0100041638). Within this LD block six genes were located: trefoil factor *(TFF1)*, trefoil factor 2 *(TFF2)*, transmembrane protease, serene 3 *(TMPRSS3)*, ubiquitin-associated and SH3 domain-containing protein A *(UBASH3A)*, radial spoke head 1 homolog (Chlamydomonas) *(RSPH1*, and solute carrier family 37 (glucose-6-phosphate transporter), member 1 *(SLC37A1)*.

### Overlapping SNPs

Comparing the list of significant markers between DH and DJ revealed that the SNP ARS-BFGL-NGS-4939 was in common between these two breeds for Zn. This SNP marker is closely linked to the DGAT1 gene. Even though we found significant SNP markers on BTA14 in both DH and DJ for Ca, and Fe these significant SNPs did not overlap.

## Discussion

In this study a genetic analysis was performed for the mineral content in bovine milk. This knowledge is important to evaluate the possibilities to change the mineral content in the milk by selective breeding.

### Mineral concentration

The mineral concentration in the milk varied between DH and DJ, with DJ having a higher milk mineral concentration; however, the CV was comparable between the two breeds. Gaucheron [10] stated that milk mineral content is relatively constant however the present study showed that there is substantial variation for the different minerals in bovine milk. This is in line with previous results showing considerable variation in milk mineral content from Swedish and Danish herds due to primarily season, but also to breed [1,2]. Results found by van Hulzen et al. [11] also showed considerable variation for mineral content in milk of Dutch Holstein-Friesian cows caused by genetic and/or environmental and nutritional variation. In line with Bijl et al. [22], we found a relatively strong correlation between Ca, Mg and P and protein, which is likely to contribute to the higher contents of these minerals in Jersey milk, as shown in the present study. This relationship is due to the association of these minerals with the casein micelle, which is also known to be of utmost importance for casein micelle stability [9,10]. Thereby their concentrations also affect the technological properties of the milk and lower Ca levels (and to some extent lower levels of P and Mg) have been associated with poor or non-coagulating milk [23-25]. Milk contents of Cu, Fe, Mn, Se and Zn further tended to be higher in DJ as compared to DH, which is in accordance with Hermansen et al. [1]. The Cu concentration in milk is known to vary between cows and with diet and level of mineral supplementation [26]. Previously it has been shown that the Cu concentration plays an important role in the spontaneous development of oxidative off-flavor of the milk [27,28]. The mineral contents reported here were based on skimmed milk, which could have affected the reported levels, as small amounts may be associated with the milk fat fraction. For instance phospholipids in the milk fat globule membrane would not have been included, which will have an effect on the milk P level as compared to earlier studies on full milk [1,2].

### Heritability

In this study the heritabilities were estimated for ten different minerals in two different cattle breeds. High heritabilities were found for Ca and Zn in both DH and DJ. For the other minerals determined the results were less consistent, i.e. a high heritability was detected in only one of the two breeds analyzed (K (0.32), Mg (0.57) (DJ) and P (0.46) (DH)). Even though the standard errors are relatively large in our study, the results are in general in agreement with the heritability estimates presented by van Hulzen et al. [11]. They also found high heritabilities for Ca (0.57) and Zn (0.41) in first parity Dutch Holstein-Friesian cows, but also for Mg (0.60), K (0.46) and P (0.62) [11].

### Screening mineral content in milk samples at large scale

The results of our study and the study of van Hulzen et al. [11] indicated that there is genetic variation for some of the minerals. This would open up the possibility to select genetically for specific minerals in the milk. However the methods used to quantify the mineral content in dairy milk are labor intensive and expensive, and are therefore not suited to be implemented for large scale screening of milk samples from the milk control system. In the routine milk laboratories mid infra red spectroscopy has been implemented to measure fat and protein in the milk. Soyeurt et al. [29] investigated the possibility to estimate the mineral concentration in the milk based on infra red. They could develop and implement prediction models for P and Ca [29], but the development of prediction models for other minerals was more difficult (e.g. Mg, Na) or impossible in case of K [29].

### QTL and genes for mineral content

To our knowledge this is the first study to report GWAS results on the mineral content in milk. As the number of samples per population is relatively small, it is expected that only those QTL with large effect are detected. First we will focus on those QTL detected for minerals with a high heritability in one of the cattle breeds (Zn and P), and then on those QTL detected for minerals with an intermediate heritability in one of the cattle breeds (K and Cu).

### Zn

For Zn we detected a QTL in DH on BTA2 with the most significant SNP assigned to the gene (*PDIK1L*) (Figure 1). *PDIK1L* is involved in biological processes like protein serine/threonine kinase activity, ATP binding and protein phosphorylation, i.e. introducing phosphate group to a protein (http://www.uniprot.org/uniprot/Q8N165). Phosphorylation of the (casein) proteins in the milk influences the stability of the casein micelle in the milk. Even though it is not known how this gene plays a role in the regulation of the Zn concentration in the milk at this stage, *PDIK1L* could be considered as a candidate gene.

A SNP marker close to *DGAT* was among the significant markers for Zn in both DH and DJ. How *DGAT* is involved in the Zn concentration in milk is at this stage unknown, however it has been shown that low *de novo* milk fat diet can significantly increase plasma Zn and milk Zn content, whereas dietary Zn level in itself did not influence Zn concentration in milk or plasma. This indicates that the transfer of fat from diet to milk might facilitate transfer of Zn from diet to milk [30].

### P

In this study total P is measured including the bound P. However we are using skimmed milk, which could explain the relative low levels of P in the milk samples compared to the literature [11]. The most significant marker (BOVINEHD0100041584) for P is located in a LD block containing 6 genes (*TFF1, TFF2, UBASH3A, RSPH1 and SLC37A1*). Based on the physical map position of BOVINEHD0100041584 and on the biological descriptions of the genes in the LD block, *SLC37A1* is a good candidate gene. BOVINEHD0100041584 is located 10 kb downstream *SLC37A1*. In human *SLC37A1* is involved in the transport of glucose-6-phoshate and plays an important role in the blood glucose homeostasis [31]. More specifically *SLC37A1* showed phosphate linked glucose-6-phoshate antiporter activity. *SLC37A1* is part of the SLC37 family. The SLC37 family is a group of genes involved in the translocation of glucose-6-phoshate from the cytoplasm into the lumen of the ER. There glucose-6-phoshate is hydrolyzed into glucose and P. [31].

### K

Cohen-Zinder et al. [32] identified *ABCG2* at 37.4 Mb on BTA6. This gene has a major effect on milk yield and milk composition [32]. The most significant SNP marker for K is located around 46.6 Mb (Figure 2), indicating that there is little or no overlap with *ABCG2*.

### Cu

The Cu concentration in the milk varies between cows and depends on the diet and level of mineral supplementation [26]. Previously it has been shown that the Cu concentration plays an important role in the spontaneous development of oxidative off-flavor of the milk [27]. QTL regulating the Cu concentration in the milk may be of interest to reduce or increase the Cu concentration by genetics. Morris et al. [33] had detected QTL for Cu on BTA3, BTA5 and BTA13. These chromosomes were also among our results for Cu content in the milk in DH. A closer look showed however that these QTL [33] did not show overlap with the significant markers for Cu content in milk detected in our study. With regard to the development of oxidative off-flavor in the milk the QTL on BTA14 for Cu is interesting. The significant SNP markers for Cu content in the milk are in LD with *DGAT1* (data not shown) i.e. the LD block was based on the HD SNP markers covering the physical map position of *DGAT1*. Juhlin et al. [28] showed that *DGAT1* polymorphisms together with the FA composition and Cu concentration were risk factors for the development of spontaneous oxidized flavor. In their study they tested the influence of *DGAT1* on different fatty acid groups, however not on the Cu concentration in the milk. Our results suggest that the *DGAT1* polymorphism may also influence the Cu concentration in the milk.

## Conclusion

Profound differences in mineral concentration in the milk between DH and DJ were identified with a generally higher mineral concentration in milk from DJ. High heritability estimates were found for Ca and Zn in both DH and DJ. The GWAS identified QTL in both DH and DJ. A QTL on BTA14 for Ca and Zn was identified in both DH and DJ. However, the majority of the QTL identified were breed specific. The relatively high heritability estimates for content of several bovine milk minerals open up the possibility of elevating milk mineral content by genetic selection.

### Availability of supporting data

No new SNPs were discovered in this manuscript. The SNPs used in this manuscript are from the Illumina Bovine HD SNP array: http://res.illumina.com/documents/products/datasheets/datasheet_bovinehd.pdf. Names and location of these SNPs can be found at: http://support.illumina.com/downloads.html. Assigning the SNPs to genes was based on the publicly available bovine genome assembly: ftp://ftp.cbcb.umd.edu/pub/data/assembly/Bos_taurus/Bos_taurus_UMD_3.1/annotation/UMD3.1.gff.gz.

## Additional files

Additional file 1: Table S1. Phenotypic correlation of between milk production traits and the mineral content of the milk. Above the diagonal the phenotypic correlation for the Danish Holstein and Danish Jersey breeds. Below the diagonal the standard error. Additional file 2: Table S2. Significant SNP associated to Mineral traits in the Danish Holstein. A genome wide association scan was performed on the Danish Holstein cattle for detailed mineral composition. SNPs were considered significant at FDR < 0.10. Additional file 3: Table S3. Significant SNP associated to Mineral traits in the Danish Jersey. A genome wide association scan was performed on the Danish Jersey cattle for detailed mineral composition. SNPs were considered significant at FDR < 0.10.
